# Hydatidiform Mole Presents As Pregnancy in a 48-Year-Old Perimenopausal Female: A Case Study

**DOI:** 10.7759/cureus.22291

**Published:** 2022-02-16

**Authors:** Farage Ftiha, Maria Levada, Yakubmiyer Musheyev, Iana Garrick, Matthew Jiang, Habiba Ahasan

**Affiliations:** 1 Medicine, New York Institute of Technology College of Osteopathic Medicine, Old Westbury, USA; 2 Obstetrics and Gynaecology, New York Institute of Technology College of Osteopathic Medicine, Old Westbury, USA

**Keywords:** benign, metastatic, human chorionic gonadotropin, gestational trophoblastic disease, perimenopausal, pregnancy, hydatidiform mole

## Abstract

Pregnancy should be suspected whenever a woman in her childbearing years misses a menstrual period. Clinical suspicion is increased if she also reports any sexual activity while not using contraception or is inconsistent in her use of contraception. Laboratory findings that aid in the diagnosis of pregnancy include the detection of human chorionic gonadotropin (hCG) in blood or urine. Hydatidiform mole (HM) is part of a group of diseases classified under gestational trophoblastic disease (GTD), which originate in the placenta and have the potential to locally invade the uterus and metastasize. Although molar pregnancies are designated as benign, they have the potential to develop into a malignancy. In this case study, we present a 48-year-old peri-menopausal female patient, with a 1+ year history of irregular menses, who presented to the clinic with signs and symptoms of pregnancy, unprotected sexual activity, and a positive at-home pregnancy test. Upon further workup of the patient, it was diagnosed that the patient had a hydatidiform molar pregnancy. It is interesting to note that benign gestational trophoblastic diseases generally occur in younger women, of “reproductive age” (generally in their twenties to early thirties), and is extremely rare in peri- and post-menopausal women.

## Introduction

The diagnosis of early pregnancy is based primarily upon laboratory assessment of human chorionic gonadotropin in urine or blood. Amenorrhea is the cardinal presenting symptom of early pregnancy. Pregnancy should be suspected whenever a woman in her childbearing years misses a menstrual period. Clinical suspicion is increased if she also reports any sexual activity while not using contraception or with inconsistent use of contraception [1].

Cessation of menses can be a difficult symptom to evaluate because some women have irregular menstrual cycles and many women have occasional prolongation of a cycle. Furthermore, vaginal bleeding/spotting is relatively common in early normal pregnancy and often occurs at or near the time that a menstrual period would be expected [2,3].

The most common signs and symptoms of early pregnancy are: amenorrhea, nausea with or without vomiting, breast enlargement and tenderness, increased frequency of urination without dysuria, and fatigue. Additional signs and symptoms may include: mild uterine cramping/discomfort without bleeding, abdominal bloating, constipation, heartburn, nasal congestion, shortness of breath, food cravings and aversions, mood changes, lightheadedness, spider angiomas, palmar erythema, increased skin pigmentation (face, linea alba, areola), difficulty sleeping, lower back pain, adnexal discomfort [1].

As mentioned previously, laboratory findings of pregnancy include detection of human chorionic gonadotropin (hCG) in blood or urine. hCG is secreted into the maternal circulation after implantation, which may occur as early as 6 days after ovulation but typically occurs eight to ten days after ovulation [4-7]. This is the earliest that hCG can be detected with a standard serum hCG test. However, the ovulation-to-implantation interval has been observed to vary by up to six days in naturally conceived pregnancies [4]. The hCG concentration doubles every 29 to 53 hours during the first 30 days after implantation of a viable, intrauterine pregnancy [1].

The diagnosis of pregnancy is based on any of the following diagnostic criteria: detection of hCG in blood or urine, identification of pregnancy by ultrasound examination, identification of fetal cardiac activity by Doppler ultrasound [1]. The number of days after the last menstrual period (LMP) before a pregnancy test becomes positive depends on several factors, including [8, 9]: cycle length, which varies because of the length of the follicular phase, and thus the timing of ovulation, varies by three to five days or more from cycle to cycle; the hCG assay's detection limit (ability to measure hCG at levels below 2 milli-international units/mL) and reference range cutoff (ie, the threshold for a positive test), which differ for serum versus urine tests; the hCG assay's combination of antibodies to hCG isoforms.

Pregnancy tests can be performed on urine or serum. Factors that influence the choice of a urine or serum pregnancy test include duration of missed menses, need for accuracy, convenience, and cost. Tests on urine are adequate for the diagnosis of a suspected pregnancy in women who have missed a menstrual period, especially when there is time to follow an initial negative test with a second test a week later. Because urine tests do not detect very low levels of hCG that would be detected by a serum test, a urine test may be negative and the serum test positive around the time of missed menses [1]. Causes of a false positive test: modern immunoassays for hCG, whether in urine or serum, specifically bind to the beta-subunit of hCG, thus preventing cross-reaction with subunits of other hormones, such as luteinizing hormone, follicle-stimulating hormone, and thyrotropin. False-positive pregnancy tests are rare and due to: operator error in performing or interpreting the test; biochemical pregnancy; exogenous hCG administered as part of infertility treatment or for athletic performance (exogenous hCG should be cleared by two weeks postinjection); hCG secretion from a tumor; pituitary hCG secretion, typically in perimenopausal and perimenopausal women; interference with the assay by anti-animal antibodies, anti-hCG antibodies, or other substances; familial hCG syndrome (a very rare genetic condition) [1].

Hydatidiform mole (HM) is part of a group of diseases classified as gestational trophoblastic disease (GTD), which originate in the placenta and have the potential to locally invade the uterus and metastasize [10]. Molar pregnancies, although benign, are considered to be premalignant because they have the potential to develop into a malignancy. Malignant disease is referred to as gestational trophoblastic neoplasia (GTN); the histologic entities included in this group are: invasive mole, choriocarcinoma, placental site trophoblastic tumor (PSTT), epithelioid trophoblastic tumor (ETT) [10].

## Case presentation

A 48-year-old peri-menopausal female Gravida 2 Para 2 presented to her ObGyn physician with complaints of a possible pregnancy and irregular periods. She was employed as an accountant. Her family history was significant for a father who had leukemia and a mother who had hypertension. Her social history revealed nothing significant but her gynecological history revealed that her last menstrual period was approximately 3 months prior. The patient was sexually active and did not have regular monthly menses in the preceding year. The patient was worried because she had a positive home pregnancy test and also admitted she has nausea, but no spotting. She did not want to be pregnant at this time.

The differential diagnosis for a middle-aged woman with nausea, missed menstrual periods and a positive pregnancy test is intrauterine pregnancy.

Transvaginal and transabdominal pelvic sonograms were performed. They showed that the endometrium was extremely thickened (32.2 mm) and heterogeneous, but no gestational sac. Further evaluation, such as advanced imaging, was recommended to exclude underlying neoplasm. Blood work revealed an hCG level of 242,296 mlU/mL, consistent with a 12 weeks pregnancy. It is significant to note that other indications of a 12-week pregnancy, such as a fetus with a heartbeat on ultrasonography were missing. As there was no viable pregnancy noted, the patient was referred for computed tomography (CT) scan of the abdomen and pelvis with contrast. The results revealed the endometrial stripe had further increased (over the span of 1 week), to 4.0 cm AP (anteroposterior) thickness, and contained multiple brightly enhancing foci, consistent with the presumed diagnosis of gestational trophoblastic disease. 

**Figure 1:**
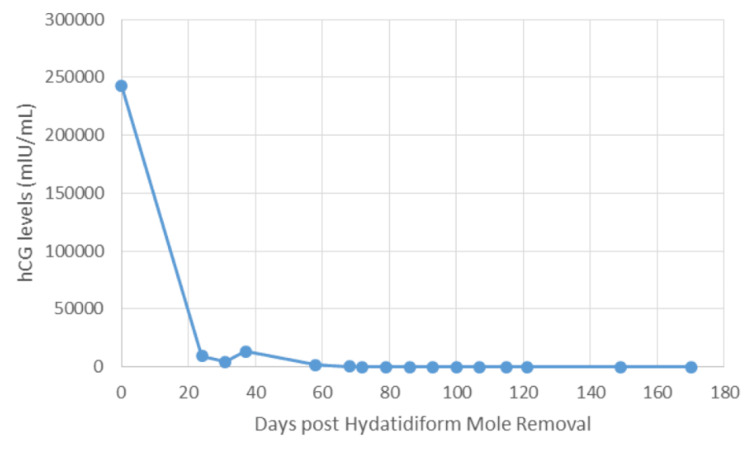
Graph 1 : hCG levels measured pre and post initial suction and sharp curettage for Hydatidiform mole Removal

The patient underwent a suction and sharp curettage under general anesthesia to evacuate the product of conception. The specimens were sent to pathology for analysis and a partial hydatidiform mole was recovered along with the aborted products. A (partial) hydatidiform mole is a placental pathology that presents as fluid-filled sacs in conjunction with a nonviable embryo. It is a benign form of gestational trophoblastic neoplasia. The partial moles were approximately 12 x 9 x 4.5 cm consisting of aggregate hemorrhagic, tan-brown soft tissue fragments in conglomerate with blood clots and vesicles. After this first surgery, hCG levels were monitored weekly, and overall there was a decreasing hCG level every week, until day 37 after the first suction and sharp curettage when hCG levels spiked up again (Figure 1). This spike ultimately led to a second follow up suction and sharp curettage in order to retrieve any remaining tissue in the uterus. All specimens were sent to pathology. Final diagnosis: blood clots, secretory endometrium, smooth muscle fibers, fragments of molar and trophoblastic tissue and benign ectocervical tissue. Specimen collected was 0.5 x 5.0 x 2.5 cm of hemorrhagic and spongy soft tissue. The final diagnosis: molar pregnancy. An addendum diagnosis of complete hydatidiform mole was given: this diagnosis was obtained following DNA index ploidy. It is interesting to note that the basis of genetic testing (via DNA index ploidy) is due to the fact that a complete hydatidiform mole is associated with having two paternal genomes with no maternal contribution [11]. In short, the diagnosis of partial hydatidiform mole was changed to complete hydatidiform mole on the basis of genetic and pathological testing.

The initial therapy for a hydatidiform mole is surgical removal. Following removal, hCG levels are monitored weekly. For this patient, Initial therapy post-operation was prophylactic methotrexate 15 mg tablet for 5 days in order to ensure that the pregnancy does not continue. However, the patient’s beta subunits hCG levels started to rise 37 days post-operation and a second suction and sharp curettage needed to be performed.

## Discussion

Patients with HM typically present to their obstetric clinician with missed menstrual periods, a positive pregnancy test, and signs and symptoms consistent with early pregnancy or early pregnancy complications (bleeding, pelvic discomfort, hyperemesis gravidarum). Molar pregnancy may be suspected based on unusually hCG levels or only after pathology evaluation of a failed pregnancy [1, 12-15].

Common early presenting symptoms of HM are: vaginal bleeding, pelvic pressure or pain, an enlarged uterus, and hyperemesis gravidarum. However, these are also common in early “normal” pregnancy, and thus, clinicians most often initially do not suspect an abnormal pregnancy complication. A diagnosis of spontaneous abortion (miscarriage) or ectopic pregnancy is much more likely, rather than HM [10].

The possibility of HM, however, should also be considered in any reproductive-age female with abnormal vaginal bleeding. A quantitative serum hCG level should be obtained and, if elevated, an ultrasound examination should be performed to correlate hCG levels with gestational age. If the clinical presentation suggests HM and the ultrasound exam confirms it, malignant gestational trophoblastic neoplasia (GTN), must be excluded. Patients should be asked about symptoms of metastatic disease and appropriate diagnostic studies (chest X-ray for one) should be done. The lungs (symptoms include dyspnea or chest pain) and vagina (vaginal bleeding) are the most common metastatic sites, but other potential sites include the central nervous system or liver [10].

To reiterate, the shared symptoms between pregnancy and hydatidiform mole include vaginal bleeding/spotting, mild uterine cramping/discomfort as well as nausea/vomiting and hyperemesis. Both pregnancy and molar pregnancy cause elevated levels of hCG. This overlap in clinical and laboratory findings may make it arduous for a clinician to differentiate between the two diagnoses.

This is why it is important for any clinician to keep an open mind when seeing patients who present with high levels of hCG, as well as the shared symptoms previously mentioned. The primary concern of molar pregnancy is that this premalignancy may evolve into a metastatic malignancy that can affect the lungs, vagina, central nervous system and liver. Because of this, symptoms related to these respective organs must be gleaned from the patient's history. In developed countries, the incidence of complete hydatidiform mole is approximately 1-3 per 1000 pregnancies and those of the partial hydatidiform mole about 3 per 1000 pregnancies [16]. 

It is also important to note that Ova from older patients may be more susceptible to abnormal fertilizations that result in this phenomenon. In fact, the risk for a complete mole was increased twofold for patients >35 years and 7.5-fold for patients >40 years [17]. Patients aged 40 or older with hydatidiform mole formation may also have a higher rate of severe complications. 

Additionally, given the above case presentation, it is interesting to note that benign gestational trophoblastic disease generally occurs in women of reproductive age and is extremely rare in perimenopausal women [18]. Because of this, the rare occurrence of hydatidiform mole in this perimenopausal patient is of the utmost interest. Furthermore, in this specific case even though this molar pregnancy was non-viable, this 48-year-old perimenopausal female is now classified as Gravida 3 Para 2.

## Conclusions

Ultimately, in this case study, we present a 48-year-old perimenopausal female patient that presented to the clinic with signs and symptoms of a normal pregnancy. Upon further workup of the patient, it was determined that the patient had a rare and potentially malignant complete hydatidiform mole. This disease has a 0.1% chance of occurrence in pregnancies, which makes this case quite unique.

## References

[1] Bastian AL Haywood LB (2021). Clinical manifestations and diagnosis of early pregnancy. UpToDate.

[2] Ananth CV, Savitz DA (1994). Vaginal bleeding and adverse reproductive outcomes: a meta-analysis. Paediatr Perinat Epidemiol.

[3] Harville EW, Wilcox AJ, Baird DD, Weinberg CR (2003). Vaginal bleeding in very early pregnancy. Hum Reprod.

[4] Wilcox AJ, Baird DD, Weinberg CR (1999). Time of implantation of the conceptus and loss of pregnancy. N Engl J Med.

[5] Braunstein GD, Rasor J, Adler D, Danzer H, Wade ME Serum human chorionic gonadotropin levels throughout normal pregnancy. Am J Obstet Gynecol.

[6] Lenton EA, Neal LM, Sulaiman R (1982). Plasma concentrations of human chorionic gonadotropin from the time of implantation until the second week of pregnancy. Fertil Steril.

[7] Lenton EA, Hooper M, King H, Kumar A, Monks N, Verma S, Osborn J (1991). Normal and abnormal implantation in spontaneous in-vivo and in-vitro human pregnancies. J Reprod Fertil.

[8] Cole LA, Ladner DG, Byrn FW (2009). The normal variabilities of the menstrual cycle. Fertil Steril.

[9] Sturgeon CM, Berger P, Bidart JM, Birken S, Burns C, Norman RJ, Stenman UH (2009). Differences in recognition of the 1st WHO international reference reagents for hCG-related isoforms by diagnostic immunoassays for human chorionic gonadotropin. Clin Chem.

[10] Berkowitz RS, Horowitz NS, Elias KM (2021). Hydatidiform mole: epidemiology, clinical features, and diagnosis. UpToDate.

[11] Carey L, Nash BM, Wright DC (2015). Molecular genetic studies of complete hydatidiform moles. Transl Pediatr.

[12] Berkowitz RS, Goldstein DP (1996). Chorionic tumors. N Engl J Med.

[13] Soto-Wright V, Bernstein M, Goldstein DP, Berkowitz RS (1995). The changing clinical presentation of complete molar pregnancy. Obstet Gynecol.

[14] Mosher R, Goldstein DP, Berkowitz R, Bernstein M, Genest DR (1998). Complete hydatidiform mole. Comparison of clinicopathologic features, current and past. J Reprod Med.

[15] Zalel Y, Dgani R (2020). Gestational trophoblastic disease following the evacuation of partial hydatidiform mole: a review of 66 cases. Eur J Obstet Gynecol Reprod Biol.

[16] Seckl MJ, Sebire NJ, Berkowitz RS (2010). Gestational trophoblastic disease. Lancet (London.

[17] Parazzini F, La Vecchia C, Pampallona S (1986). Parental age and risk of complete and partial hydatidiform mole. Br J Obstet Gynaecol.

[18] Garcia M, Romaguera RL, Gomez-Fernandez C (2004). A hydatidiform mole in a postmenopausal woman. A case report and review of the literature. Arch Pathol Lab Med.

